# Cellular senescence induced by aberrant MAD2 levels impacts on paclitaxel responsiveness *in vitro*

**DOI:** 10.1038/sj.bjc.6605419

**Published:** 2009-11-24

**Authors:** M Prencipe, P Fitzpatrick, S Gorman, M Mosetto, R Klinger, F Furlong, M Harrison, D O'Connor, I B Roninson, J O'Sullivan, A McCann

**Affiliations:** 1 UCD School of Medicine and Medical Science (SMMS), University College Dublin, UCD, Belfield, Dublin 4, Ireland; 2 UCD School of Public Health and Population Science, University College Dublin, UCD, Belfield, Dublin 4, Ireland; 3 Centre for Colorectal Disease, St Vincents University Hospital, Dublin 4, Ireland; 4 School of Biomolecular and Biomedical Science (SBBS), University College Dublin, UCD, Belfield, Dublin 4, Ireland; 5 Department of Pathology, Mater Misericordiae Hospital, Eccles Street, Dublin 7, Ireland; 6 Cancer Centre, Ordway Research Institute, Albany, NY, USA

**Keywords:** mitotic assembly deficient protein 2 (MAD2), cellular senescence, paclitaxel resistance, breast cancer

## Abstract

**Background:**

The mitotic arrest deficiency protein 2 (MAD2) is a key component of the mitotic spindle assembly checkpoint, monitoring accurate chromosomal alignment at the metaphase plate before mitosis. MAD2 also has a function in cellular senescence and in a cell's response to microtubule inhibitory (MI) chemotherapy exemplified by paclitaxel.

**Methods:**

Using an siRNA approach, the impact of MAD2 down-regulation on cellular senescence and paclitaxel responsiveness was investigated. The endpoints of senescence, cell viability, migration, cytokine expression, cell cycle analysis and anaphase bridge scoring were carried out using standard approaches.

**Results:**

We show that MAD2 down-regulation induces premature senescence in the MCF7 breast epithelial cancer cell line. These MAD2-depleted (MAD2↓) cells are also significantly replicative incompetent but retain viability. Moreover, they show significantly higher levels of anaphase bridges and polyploidy compared to controls. In addition, these cells secrete higher levels of IL-6 and IL-8 representing key components of the senescence-associated secretory phenotype (SASP) with the ability to impact on neighbouring cells. In support of this, MAD2↓ cells show enhanced migratory ability. At 72 h after paclitaxel, MAD2↓ cells show a significant further induction of senescence compared with paclitaxel naive controls. In addition, there are significantly more viable cells in the MAD2↓ MCF7 cell line after paclitaxel reflecting the observed increase in senescence.

**Conclusion:**

Considering that paclitaxel targets actively dividing cells, these senescent cells will evade cytotoxic kill. In conclusion, compromised MAD2 levels induce a population of senescent cells resistant to paclitaxel.

Cellular senescence can be described as replicative or premature. Telomere shortening results in the former (Harley *et al*, 1990; Bodnar *et al*, 1998), whereas oncogene activation (e.g. mutations in K-ras, B-raf, PTEN and NF1) (Courtois-Cox *et al*, 2008) and stress signals (Shay and Roninson, 2004) such as DNA targeting chemotherapy (doxorubicin or cisplatin) or microtubule inhibitory (MI) chemotherapy (paclitaxel and vincristine) result in the latter. Typically, senescent cells acquire a characteristic flattened and enlarged morphology. In addition, the cytoplasm appears vacuole-rich and granulated due to the expansion of the lysosomal compartment leading to increased expression of the lysosomal *β*-galactosidase enzyme, a commonly assessed marker of senescence. These cells also become refractory to apoptosis (Hampel *et al*, 2004) displaying an altered gene expression profile, with the down-regulation of genes involved in cell proliferation (e.g. Ki-67 and CDC2) and up-regulation of multiple genes with documented growth-inhibitory activity (e.g. p16) (Chang *et al*, 2002).

Recently, substantial evidence has identified cellular senescence as an alternative tumour suppressor mechanism to apoptosis (Courtois-Cox *et al*, 2008). Supporting this function, following oncogene activation, senescent cells can be found in pre-malignant lesions but not in malignant tumours (Collado *et al*, 2005), identifying an involvement of cellular senescence in preventing tumour progression. However, their presence may also identify a more sinister function in that senescent cells can interfere with their microenvironment, by secreting proteases and mitogenic, antiapoptotic and angiogenic factors, which may promote carcinogenesis in neighbouring cells (Chang *et al*, 2002). This phenomenon, recently described as the ‘senescence-associated secretory phenotype’ (SASP) (Young and Narita, 2009), is gaining considerable research interest. In addition, although it has been documented that cellular senescence is irreversible (Hayflick, 1965), several reports refute this (Beauséjour *et al*, 2003; Rajaraman *et al*, 2006). Specifically, Beauséjour *et al* (2003) have shown that it is possible to revert cellular senescence following inactivation of p53, if p16 is concomitantly down-regulated.

The mitotic arrest deficiency protein 2 (MAD2) is an essential component of the mitotic spindle checkpoint pathway and has a crucial function in the transition from metaphase to anaphase by delaying anaphase onset, through inhibition of the anaphase promoting complex/cyclosome (APC/C). This ensures that all chromosomes are correctly aligned at the metaphase plate prior to daughter cell segregation (Wang *et al*, 2002). For this reason, a compromised mitotic spindle assembly checkpoint has an important function in chromosomal instability (CIN) (Yoon *et al*, 2002), characterised by accelerated rates of chromosomal aberration (Lengauer *et al*, 1998) and resultant aneuploidy (Lengauer *et al*, 1997). Although the molecular mechanisms underlying a defective mitotic spindle assembly checkpoint and associated CIN in cancer remain elusive, it is notable that cancer cell lines expressing low levels of MAD2 display a defective mitotic checkpoint concomitant with CIN (Li and Benezra, 1996; Percy *et al*, 2000). Importantly, MAD2 has a crucial function in the malignant transformation of epithelial cells, promoting aneuploidy and predisposing human prostate epithelial cells to carcinogen-induced malignant transformation (To-Ho *et al*, 2008). This suggests that inactivation of the mitotic spindle assembly checkpoint, through aberrant expression of MAD2, may constitute one of the very initial steps in the malignant progression of human cancer.

Recently, To-Ho *et al* (2008) have shown a relationship between the cellular expression of a truncated form of the MAD2 protein and increased senescence in human prostate epithelial cells. MAD2 has also been shown to be down-regulated in doxorubicin-induced senescent cells (Chang *et al*, 2002). Because of its integral function as a cell cycle checkpoint during mitosis, MAD2 is also thought to have a function in paclitaxel resistance (Sudo *et al*, 2004).

As a chemotherapeutic agent, paclitaxel is increasingly being used as a MI chemotherapy treatment for breast, ovarian and other solid cancers. However, up to a third of patients are not responsive and although several mechanisms have been associated with paclitaxel resistance (McGrogan *et al*, 2008), reliable molecular markers predicting resistance remain elusive.

In this study, we show a significant association between depleted levels of MAD2 and the induction of cellular senescence, with further senescence induction following paclitaxel treatment in human breast cancer cells. We suggest that MAD2-depleted tumours will not be eradicated by MI therapy exemplified by paclitaxel due to the presence and further induction of a viable senescent cell population capable of interfering with and impacting on the microenvironment through the secretion of transforming growth factors that influence their neighbouring cells (Young and Narita, 2009). Moreover, senescent cells also have the potential to re-enter the cell cycle identifying them as highly chemo-refractory (Beauséjour *et al*, 2003; Rajaraman *et al*, 2006).

## Materials and methods

### Cell culture and treatments

MCF-7 cells (ATCC) are an epithelial-like breast adenocarcinoma cell line. Routinely, they were cultured in DMEM medium (Gibco, Carlsbad, CA, USA) supplemented with 10% fetal bovine serum, 100 *μ*l ml^−1^ streptomycin and 100 U ml^−1^ penicillin. HT1080 human fibrosarcoma cells (ATCC) were cultured in MEM medium (Gibco) supplemented with 10% fetal bovine serum, 100 *μ*l ml^−1^ streptomycin and 100 U ml^−1^ penicillin. Derivation of the HT1080 p21-9 cell line carrying p21 in an isopropyl-*β*-D-thiogalactoside (IPTG)-inducible retroviral vector has been described earlier (Chang *et al*, 1999). The paclitaxel experiments consisted of two groups of cells, one untreated and the other treated with 100 nM paclitaxel (Sigma-Aldrich, St Louis, MO, USA) over a 72-h period. The IPTG induction of p21 was achieved by treating the cells with 50 *μ*M IPTG (Fisher Scientific, Hampton, NH, USA) for 3 days.

### Small-interfering RNA transfection

Small-interfering RNA (siRNA) duplexes targeting MAD2 were purchased from Ambion (Austin, TX, USA). MCF7 transfection was performed with Lipofectamine 2000 following commercial (Invitrogen, Carlsbad, CA, USA) protocols using a 40 nM final concentration of siRNA. Cells transfected with scramble siRNA duplexes (40 nM Ambion) controlled for any off-target effect of siRNA transfection.

### Western blot analysis

Cells were harvested and washed in PBS and lysed in RIPA lysis buffer (Sigma) containing protease and phosphatase inhibitors. Resultant lysates were cleared by centrifugation at 14 000 rpm for 20 min at 4°C. Thirty *μ*g of protein were resolved by SDS–polyacrylamide gel electrophoresis and transferred to a nitrocellulose membrane (Amersham, Little Chalfont, UK), which was subsequently blocked in 5% milk and probed with the anti-MAD2 antibody (1 : 500, BD Biosciences), anti-Cyclin B1 (1 : 2000, Cell Signaling Technology, Danvers, MA, USA) or p21 (1 : 1000, Cell Signaling Technology) overnight at 4°C. *β*-actin antibody (1 : 2000, Sigma-Aldrich) or alternatively GAPDH (1 : 1000, Chemicon International, Temecula, CA, USA), were used as loading controls. The membrane was rinsed in TBS Tween20 and incubated with horseradish peroxidase-conjugated anti-mouse secondary antibody (Pearse, Waltham, MA, USA) and visualised by an Enhanced Chemiluminescence detection system (Pearse).

Densitometrical analysis was carried out using Scion image analysis (http://www.scioncorp.com), following loading control (*β*-actin) adjustment.

### Senescence-associated *β*-galactosidase activity

One hundred and fifty thousand cells were seeded in six-well plates. Following MAD2 depletion and paclitaxel treatment for 72 h, the cells were washed twice with PBS, fixed with 2% formaldehyde (Sigma) and 0.2% glutaraldehyde (Sigma-Aldrich) in water for 10 min and washed again in 2 PBS washes. Cells were stained with X-gal staining solution (1 mg ml^−1^ X-gal, 40 mmol l^−1^ citric acid/sodium phosphate pH 6.0, 5 mmol l^−1^ potassium ferricyanide, 5 mmol l^−1^ potassium ferrocyanide, 150 mmol l^−1^ NaCl, 2 mmol l^−1^ MgCl_2_) for 24 h in a CO_2_ incubator at 37°C. The cells were subsequently rinsed in two washes of PBS and counted using a phase contrast microscope (Olympus CK2, Shinjuku, Tokyo, Japan). Senescent cells were expressed as a percentage of the total number of cells counted (300 cells per well).

### Colony forming assay

Following MAD2 depletion and paclitaxel treatment, cell proliferation was assessed as described by Franken *et al* (2006). This assay allows enumeration of the number of colonies formed from a single cell seeded. Briefly, after transfection and drug treatment for 72 h, the cells were trypsinized and 500 cells per well were seeded in six-well plates. After 10 days, the media was removed, the cells were washed in two PBS washes and fixed/stained with a mixture of 6.0% glutaraldehyde (Sigma-Aldrich) and 0.5% crystal violet (Avonchem, Macclesfield, UK) solution in water for 30 min at room temperature. The plates were then rinsed in tap water and left to dry overnight. The colonies were counted and the plate efficiency was calculated as the number of colonies formed/number of cells seeded × 100%.

### Cell viability assay

Ten thousand MCF-7 cells per well were cultured in a 96-well plate and transfected with siRNA targeting MAD2, scramble siRNA or left untransfected. Twenty-four hours after transfection, the cells were either treated with 100 nM of paclitaxel or untreated. Ten thousand HT1080 p21-9 cells per well were cultured in a 96-well plate and either treated with 50 *μ*M IPTG for 3 days or left untreated. Subsequently, the two groups of cells (−/+IPTG) were either treated with 100 nM of paclitaxel or left untreated.

After 72 h, the effect on cell viability was examined by the 3-(4,5)-dimethylthiazol-2-yl-2,5-diphenyltetrazolium bromide (MTT) assay. Fifty *μ*l of 3-(4,5)-dimethylthiazol-2-yl-2,5-diphenyltetrazolium bromide solution (5 mg ml^−1^ in PBS; Sigma-Aldrich) were added to each well and the cells were incubated in a CO_2_ incubator at 37°C for 5 h. Following media removal, the 3-(4,5)-dimethylthiazol-2-yl-2,5-diphenyltetrazolium bromide-formazan formed by metabolically viable cells was dissolved in 200 *μ*l of DMSO (Sigma-Aldrich), and the absorbance was measured in a plate reader at 550 nm.

### Flow cytometric analysis of the cell cycle

Cell cycle distribution was measured both in the untreated and paclitaxel treated cells. Cells were harvested after 72 h of treatment, washed in PBS and fixed for at least 4 h in 70% cold methanol. The pellets were then washed twice in PBS Tween20 and resuspended in PBS. Ribonuclease A (0.2 mg ml^−1^, Sigma) was added, and the cells were incubated at 37°C for 30 min. Propidium iodide (50 *μ*g ml^−1^, Sigma) was added and the samples were kept on ice for 30 min before fluorescence-activated cell sorting (FACS) analysis. Data from 10 000 cells were collected. Flow cytometry data were analysed by Summit 4.2 software (Dako Colorado, Inc., Fort Collins, CO, USA).

### Anaphase bridge scoring

MCF-7 cells untransfected or transfected with scramble siRNA or MAD2 siRNA were fixed in 70% methanol and visualised using haematoxylin staining, which is a nuclear staining, before anaphase bridge enumeration. The number of bridges was expressed as a percentage over the total number of cells counted (*n*=2000).

### Invasion assay

A total of 50 000 cells were incubated in the upper chamber of an innocyte migration assay well (Becton Dickinson, Franklin Lakes, NJ, USA) and allowed to migrate through an 8-mm pore for 24 h. Migrated cells were detached using a detachment buffer provided with the kit and their number was quantified using a cell-permeable fluorescent dye. Fluorescence was read on a plate reader at excitation and emission wavelengths of 485 nm and 520 nm, respectively. The number of migrated cells was normalised to cell growth levels.

### Quantification of pro-inflammatory cytokines using the MSD multi-spot human cytokine assays

The levels of interleukin-8 (IL-8), IL-1*β* and IL-6 were measured in tissue culture media using a four-spot multi-array plate (Meso Scale Discovery, Gaithersburg, Washington DC, USA). The assay was carried out following the protocol provided and read using the Sector Imager 2400. The levels of secretions for each cytokine were normalised to cell proliferation.

### Statistical analyses

Where assumptions could be met, analysis of variance with bonferroni testing for inter-group comparisons was used for the comparison of means in three groups; if not, non-parametric tests were used. The Wilcoxon rank sum test was used for two group comparisons. The SAS programme was used for these analyses.

## Results

### MAD2 down-regulation induces cellular senescence in MCF7 breast cancer cells

**Figure 1:**
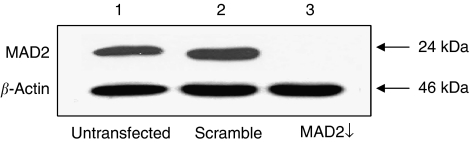
siRNA MAD2 protein down-regulation in MCF-7 breast cancer cells. Western blot analysis demonstrates a robust reduction of the MAD2 protein in the MAD2↓ cells (lane 3) compared with the untransfected (lane 1) and scramble (lane 2) controls. *β*-actin was used as a loading control.

**Figure 2:**
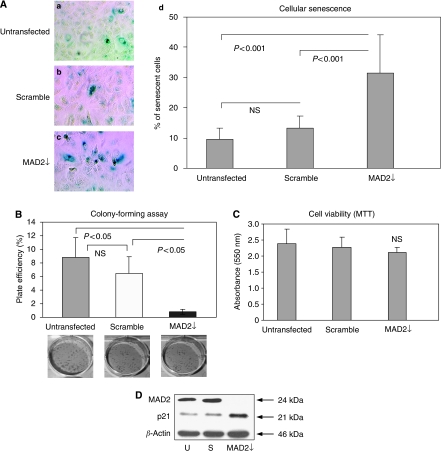
MAD2 down-regulation induces cellular senescence in MCF-7 breast cancer cells. (**A**) 15 × 10^4^ cells were seeded in each well of a six-well plate. They were either left untransfected or transfected with either MAD2 siRNA or scramble siRNA. Post siRNA reduction of MAD2, morphological changes were evident with MAD2-depleted (MAD2↓) cells appearing flattened and enlarged and more vacuole-rich, compared with the untransfected and scramble controls. SA-*β*-galactosidase staining (panels a–c) shows a significantly higher percentage of senescent cells in the MAD2↓ cells compared with the untransfected and scramble controls (*P*<0.001) (panel d). (**B**) Plate efficiency percentage (number of colonies formed/number of cells seeded) × 100%, in MAD2↓ cells compared with the untransfected and scramble controls. The colony count showed a significant lower number of colonies in MAD2↓ cells, compared with the controls (*P*<0.05). (**C**) The MTT assay showed the same viability for untransfected, scramble and MAD2↓ cells (*P*>0.05). (**D**) Western blotting analysis showed up-regulation of the p21 senescent marker following siRNA down-regulation of MAD2 compared with the untransfected and the scramble controls. *β*-actin was used as a loading control.

Post transfection of the human breast epithelial carcinoma cell line MCF-7 with an siRNA targeting MAD2, western blot analysis showed a robust reduction of the MAD2 protein, compared with the untransfected and scramble controls (Figure 1). Morphologically, MAD2-depleted cells (MAD2↓) appeared flattened and enlarged compared with the controls and their cytoplasm typically vacuole-rich typical of senescent cells. Senescence-associated *β*-galactosidase activity staining, confirmed that these cells were senescent (Figure 2A, panel c), with a two-fold increase in the percentage of senescence in MAD2↓ cells compared to the untransfected and scramble controls (*P*<0.001) (Figure 2A, panel d). Performance of a colony forming assay 96 h following siRNA transfection (Figure 2B) showed a significantly lower number of colonies (*P*<0.05) in MAD2↓ cells compared with the untransfected and scramble controls. However, the 3-2,5-diphenyltetrazolium bromide (MTT) viability assay (Figure 2C), showed a similar viability in MAD2↓ cells compared with the controls (*P*>0.05). The fact that MAD2 down-regulation induced replicative-compromised cells that were viable confirms that these cells had undergone cellular senescence. In addition, confirmatory western blot analysis showed up-regulation of p21, a key marker of cellular senescence (Chang *et al*, 2000), in the MAD2↓ cells compared with the untransfected and scramble controls (Figure 2D).

### MAD2 down-regulation alters cell cycle kinetics inducing anaphase bridge formation

**Figure 3:**
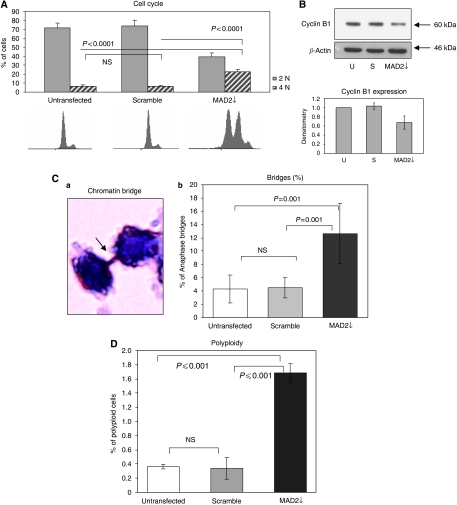
MAD2 down-regulation alters cell cycle kinetics inducing anaphase bridge formation. (**A**) Following MAD2 siRNA down-regulation, the cell cycle appeared highly de-regulated, showing less cells with 2N DNA content (*P*<0.0001) and more in 4N (*P*<0.0001). (**B**) Western blotting analysis of cyclin B1 shows a significant decrease of this protein in the MAD2↓ cells compared with the untransfected and scramble controls. (**C**) Anaphase bridges were visualised using haematoxylin staining, which is a nuclear staining able to highlight only the nuclear content (panel a). MAD2↓ cells demonstrate a three-fold higher percentage of anaphase bridges compared with the untransfected and scrambled controls (*P*⩽0.001) (panel b). (**D**) MAD2 down-regulation also results in a higher percentage of polyploid cells (>4N) than the control lines (*P*⩽0.001). Flow cytometry data were analysed by Summit 4.2 software. Mean values were compared using the *t*-test assuming equal variances.

As expected, FACS analysis showed a highly de-regulated cell cycle, with MAD2↓ cells displaying a significant increase in 4N DNA content (*P*<0.0001) compared with the scramble and untransfected controls (Figure 3A). To determine whether these were 4N cells arrested in mitosis or alternatively tetraploid G1 cells, we assessed the cyclin B1 levels by western blotting (Figure 3B), finding a decrease in cyclin B1 in MAD2↓ cells, indicative of a mitotic slippage. This points to the fact that MAD2↓ cells are not retained in mitosis long enough to allow for efficient chromosomal segregation. This suggests that, as a consequence, aberrant mitosis may occur resulting in anaphase bridge formation. These bridges represent lagging chromosomes, caused by sister chromatids failing to separate completely at the metaphase–anaphase transition. When they do eventually break, one daughter cell can end up with either a gain or a loss of chromosomal material (Saunders, 2003). Significantly in this study, anaphase bridge enumeration showed a three-fold higher percentage of anaphase bridges in the MAD2↓ cells compared with the untransfected and scramble controls (*P*⩽0.001) (Figure 3C). Moreover, we showed a significantly higher percentage of polyploidy (*P*<0.01) (Figure 3D) in the MAD2↓ cells compared with controls.

### MAD2 down-regulation alters the protein secretion of cells and enhances cell migration

**Figure 4:**
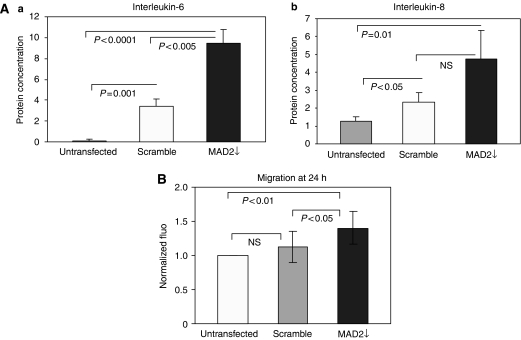
MAD2 down-regulation alters the protein secretion of cells and enhances cell migration. (**A**) (Panel a) ELISA analysis shows a three-fold increase in IL-6 levels in the MAD2↓ cells compared with the scramble (*P*<0.005) and the untransfected (*P*<0.001) controls. (Panel b) MAD2↓ cells show a higher expression of IL-8 compared with the controls. However, this increase reaches statistical significance only in comparison with the untransfected control (*P*=0.01). (**B**) MAD2↓ cells show a higher migration ability compared with the untransfected (*P*<0.01) and scramble (*P*<0.05) controls. Mean values were compared using the *t*-test assuming equal variances.

Having found a direct association between MAD2 down-regulation and cellular senescence induction and considering the known ability of senescent cells to secrete inflammatory cytokines capable of interfering with their microenvironment (Young and Narita, 2009), we performed an ELISA test to look at the expression levels of IL-1*β*, IL-6 and IL-8 following MAD2 down-regulation. Intriguingly, we have found a three-fold increase in IL-6 in the MAD2↓ cells compared with the scramble control (*P*<0.01) and nine-fold compared with the untransfected control (*P*<0.001) (Figure 4A, panel a). IL-8 expression was significantly higher in the MAD2↓ cells compared with the untransfected control (*P*=0.01) but did not reach significance in comparison with the scramble control, despite showing a trend towards higher levels in the MAD2↓ cells (*P*=0.06) (Figure 4A, panel b). Moreover, IL-1*β* did not show a statistically significant increase in MAD2↓ cells compared with the controls. Interestingly, a migration assay showed a statistically significant higher migration ability of the MAD2↓ cells compared with the untransfected (*P*<0.01) and scramble (*P*<0.05) controls (Figure 4B).

### MAD2 reduction further exacerbates the induction of viable senescent cells after paclitaxel treatment

**Figure 5:**
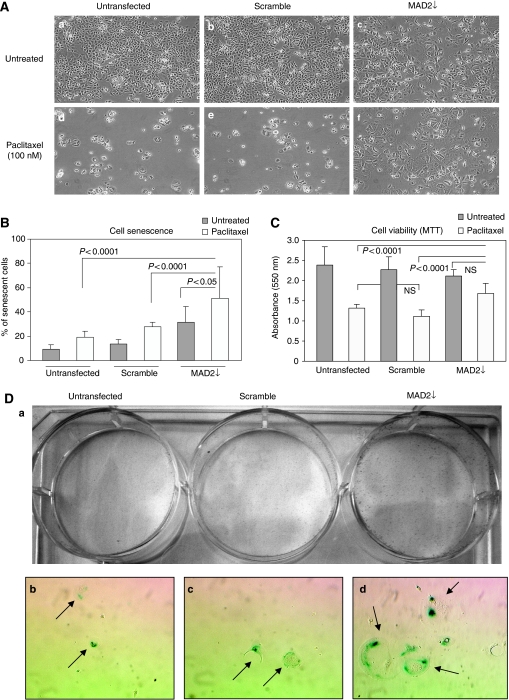
MAD2 down-regulation induces paclitaxel resistance through cellular senescence induction in MCF7 cells. (**A**) 15 × 10^4^ cells were seeded in each well of a six-well plate. They were either left untransfected or transfected with either MAD2 siRNA or scramble siRNA. Twenty-four hours following transfection, they were either treated with 100 nM of paclitaxel or left untreated. Phase contrast images captured 72 h post paclitaxel treatment showed that MAD2↓ cells remained attached to the flask with many displaying typical senescence morphology (panel f), contrasting dramatically with the untransfected and scramble controls (Panels d and e), which display a rounded shape eventually detaching from the flask. (**B**) The SA-*β*-galactosidase staining post paclitaxel treatment shows an increase in cellular senescence in the MAD2↓ cells compared with the untransfected and scramble controls (*P*<0.0001). Moreover, a significantly higher percentage of senescent cells in the MAD2↓-depleted cells was observed (*P*<0.05) compared with the MAD2↓ untreated cells. (**C**) The MTT assay shows a significantly higher (*P*<0.0001) percentage of viability in the MAD2↓ cells post paclitaxel treatment compared with the untransfected and scramble controls. (**D**) Post transfection and 100 nM paclitaxel treatment for 72 h, cells were trypsinized and 10 000 cells were seeded in each well and cultured for 10 days. (Panel a) Crystal violet staining showed significantly more viable cells in the MAD2↓ well compared with the untransfected and scramble controls. (Panels b–d) Phase contrast pictures at 10 × magnification show more senescence cells (arrows) in the MAD2↓ well compared with the untransfected and scramble controls.

Due to the observed association between MAD2 down-regulation and the induction of cellular senescence, we investigated how MAD2 depletion would impact on the MAD2↓ cells’ response to paclitaxel. Phase contrast images captured 72 h post paclitaxel treatment, showed that MAD2↓ cells remained attached to the flask with many displaying typical senescence morphology (Figure 5A, panel f). This was in stark contrast to the untransfected and scramble controls (Figure 5A, panels d and e), which displayed a rounded shape, indicative of G2/M arrest or early apoptosis (data not shown) with eventual detaching from the flask; the expected paclitaxel response. The SA-*β*-galactosidase staining following paclitaxel treatment, showed not only a significant increase in cellular senescence in MAD2↓ cells compared with the untransfected and scramble controls (*P*<0.0001) (Figure 5B), but significantly, a further induction of senescence in the paclitaxel treated MAD2↓ cells compared with the paclitaxel naive cultures (*P*<0.05). The MTT assay, showed more viable cells (*P*<0.0001) after paclitaxel, in the MAD2↓ cells compared with the untransfected and scramble controls (Figure 5C), whereas no difference in viability was displayed in the MAD2↓ cells before and after paclitaxel treatment. In support of this observation, we performed a colony forming assay after 72 h of paclitaxel treatment, seeding 10 000 cells per well for each condition (untransfected, scramble and MAD2↓). In parallel, we also seeded cells for SA-*β*-galactosidase staining. After 10 days, although there was no colony formation in any group, there were demonstrably more viable cells in the MAD2↓ well, compared with the untransfected and scramble controls (Figure 5D, panel a). Moreover, the SA-*β*-galactosidase staining displayed an increase in cellular senescence in MAD2↓ cells compared with the untransfected and scramble controls (Figure 5D, panels b–d). These results confirm that the difference in viability in the MAD2↓ cells post paclitaxel treatment, shown by both the MTT assay and by the colony forming assay, was due to senescent cells evading paclitaxel-induced death.

To further support the direct link between cellular senescence and resistance to paclitaxel, we used the human HT1080 p21-9 fibrosarcoma cell line, carrying an IPTG-induced expression cassette, able to drive p21 up-regulation. The cyclin-dependent kinase inhibitor, p21^Waf/Cip1/Sdi1^, is a key negative regulator of cell cycle progression and its up-regulation has been associated with cellular senescence induction (Chang *et al*, 2000).

**Figure 6:**
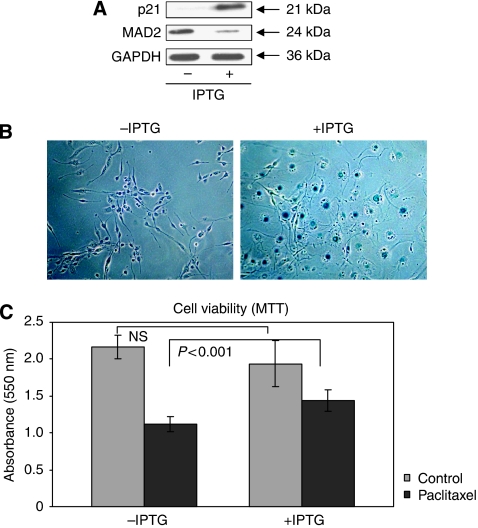
p21 induction of cellular senescence induces paclitaxel resistance in HT1080 p21-9 human fibrosarcoma cells. (**A**) Western blot analysis shows down-regulation of the MAD2 protein following up-regulation of p21, induced by IPTG. GAPDH was used as a loading control. (**B**) *β*-galactosidase staining shows an increase in senescent cells following IPTG induction of p21 and concomitant MAD2 down-regulation. (**C**) The MTT cell viability assay shows the same viability in the HT1080 p21-9 cells before paclitaxel treatment, regardless of p21 induction by IPTG. However, a significant higher viability is displayed by the IPTG-treated cells when compared with the IPTG non-treated cells, after paclitaxel (*P*<0.001).

We have shown that up-regulation of p21, in these cells, is able to induce MAD2 down-regulation (Figure 6A) and also an increase in cellular senescence (Figure 6B). An MTT assay showed no difference in viability between the HT1080 p21-9 cells with or without IPTG. This is in agreement with the fact that senescent cells show the same viability as normal cells. However, when we treated the HT1080 p21-9 cells with paclitaxel, a significant difference in viability (*P*<0.001) was detected between the cells that had been induced with IPTG to become senescent, compared with the cells that had not been IPTG induced. Specifically, the latter showed a 2.5-fold increase in cell death in comparison to the former (Figure 6C).

## Discussion

Several reports have suggested a function for MAD2 in the onset of premature senescence. Specifically, MAD2 is down-regulated in doxorubicin-induced senescent cells (Chang *et al*, 2002), its expression is reduced *in vitro* in aging pig oocytes (Ma *et al*, 2005) and the expression of a truncated form of MAD2 is associated with cellular senescence in human prostate epithelial cells (To-Ho *et al*, 2008). In this study, we have found the first link between the induction of cellular senescence and MAD2 down-regulation in human breast cancer cells. Following down-regulation of MAD2, the MCF-7 breast cancer cells underwent a dramatic change in their morphology, cell cycle and ability to proliferate; hallmarks of cellular senescence. At 72 h following MAD2 down-regulation, ∼30% of the cells showed features of senescence, a result strikingly similar to that reported by To-Ho *et al* in human prostate epithelial cells, following expression of a truncated form of MAD2 protein. These comparative results, in cells of different lineages, corroborate the hypothesis that aberrant MAD2 levels have a significant function in the induction of cellular senescence.

The induction of this form of cellular fate is biologically significant as senescent cells are able to modify their microenvironment by the secretion of transforming factors (Kuilman and Peeper, 2009; Young and Narita, 2009). Interestingly, following MAD2 down-regulation, we have shown an aberrant increased secretion of IL-6 and IL-8, which have been identified as key components of the SASP (Coppé *et al*, 2008), having a crucial function in EMT induction. Relevantly, this result was mirrored by the increased ability of migration displayed by MAD2↓ cells.

Our data also show a significant increase in anaphase bridge formation and a higher percentage of polyploid cells post MAD2 down-regulation. Significantly, as reviewed by Storchova and Pellman (2004), polyploid cells are highly unstable, potentially contributing to aneuploidy in cancer, whereas anaphase bridge formation may result in severe aneuploidy, clearly associated with CIN in cancer (Lengauer *et al*, 1997). The fact that MAD2 defective cells are more prone to CIN, through anaphase bridge formation, is particularly interesting from a treatment point of view. In fact, it has been shown that the presence of CIN in tumours before drug exposure, seems to predict for intrinsic taxane resistance (Swanton *et al*, 2006).

Following on from these observations, we investigated what effect premature senescence (induced by aberrant MAD2 levels) had on paclitaxel responsiveness. SA-*β*-galactosidase staining showed that, after paclitaxel treatment, 50% of the population had undergone senescence in the MAD2↓ cells. This percentage was higher than that seen in the paclitaxel naive MAD2↓ cells (30%), suggesting that paclitaxel had in fact exacerbated the induction of cellular senescence. We also found that there were significantly more viable cells in the MAD2↓ population after paclitaxel, reflecting the observed increase in cellular senescence. Most importantly, the fact that the colony forming assay did not show any colony formation after paclitaxel treatment for either of the three groups (untransfected, scramble and MAD2↓), is not an indication that the cellular fate was similar in each of the three groups. In fact, the colony forming assay can only give information about the ability of the cells to proliferate but cannot discriminate the cellular mechanism underlying this lack of proliferation, which in our case appears to be apoptosis in the two control groups with cellular senescence in the MAD2↓ cells.

Further support of our findings comes from the HT1080 p21-9 data, where we have shown a 2.5-fold higher viability (*P*<0.001) post paclitaxel treatment, on senescence induction through up-regulation of p21 and consequent MAD2 down-regulation.

The ominous function that senescent cells may have in the chemotherapeutic arena lies in the fact that firstly, recent evidence has shown that senescent cells can interfere with their microenvironment, by secreting transforming factors, which may promote carcinogenesis in neighbouring cells (Chang *et al*, 2002), mirrored by our cytokine data. Secondly, although it is widely documented that cellular senescence is irreversible (Hayflick, 1965), other data refute this (Beauséjour *et al*, 2003; Rajaraman *et al*, 2006). Intriguingly, Rajaraman *et al* describe a novel process of cell division occurring only in senescent cells, termed *neosis* resulting in multiple smaller cells (Raju cells), which in turn are able to re-enter the cell cycle giving rise to a more aggressive cell population.

In summary, we have shown that down-regulation of MAD2, a feature common to many tumour cell lineages (Takahashi *et al*, 1999; Wang *et al*, 2000, 2002; Fung *et al*, 2007) induces cellular senescence in up to a third of the cell population. Due to the fact that paclitaxel targets actively dividing cells, these cells will evade cytotoxic-induced death. Corroborating this, we have found no decrease in cell viability post paclitaxel treatment in the MAD2↓ cells and a significant increase in cell senescence. Our results warrant further investigation as paclitaxel treatment may not be beneficial and even deleterious to those tumours displaying low levels of MAD2.

## References

[bib1] Beauséjour CM, Krtolica A, Galomi F, Narita M, Lowe SW, Yaswen P, Campisi J (2003) Reversal of human cellular senescence: role of the p53 and p16 pathway. EMBO J 16: 4212–4222 10.1093/emboj/cdg417 PMC175806 12912919

[bib2] Bodnar AG, Ouellette M, Frolkis M, Holt SE, Chiu CP, Morin GB, Harley CB, Shay JW, Lichtsteiner S, Wright WE (1998) Extension of life span by introduction of telomerase into normal human cells. Science 279: 349–352 9454332 10.1126/science.279.5349.349

[bib3] Chang BD, Xuan Y, Broude EV, Zhu H, Schott B, Fang J, Roninson IB (1999) Role of p53 and p21waf1/cip1 in senescence-like terminal proliferation arrest induced in human tumor cells by chemotherapeutic drugs. Oncogene 18(34): 4808–4818 10490814 10.1038/sj.onc.1203078

[bib4] Chang BD, Watanabe K, Broude EV, Fang J, Poole JC, Kalinichenko TV, Roninson IB (2000) Effects of p21^Waf1/Cip1/Sdi1^ on cellular gene expression: implications forcarcinogenesis, senescence, and age-related diseases. PNAS USA 97(8): 4291–4296 10760295 10.1073/pnas.97.8.4291 PMC18232

[bib5] Chang BD, Swift ME, Shen M, Fang J, Broude EV, Roninson IB (2002) Molecular determinants of terminal growth arrest induced in tumor cells by a chemotherapeutic agent. PNAS 99: 389–394 11752408 10.1073/pnas.012602599 PMC117570

[bib6] Collado M, Gil J, Efeyan A, Guerra C, Schuhmacher AJ, Barradas M, Benguría A, Zaballos A, Flores JM, Barbacid M, Beach D, Serrano M (2005) Senescence in premalignant tumours. Nature 436: 642 16079833 10.1038/436642a

[bib7] Coppé JP, Patil CK, Rodier F, Sun Y, Munoz DP, Goldstein J, Nelson PS, Desprez PY, Campisi J (2008) Senescence-associated secretory phenotypes reveal cell-nonautonomous functions of oncogenic RAS and the p53 tumour suppressor. PLoS Biol 6: e301 10.1371/journal.pbio.0060301 PMC2592359 19053174

[bib8] Courtois-Cox S, Jones SL, Cichowsky K (2008) Many roads lead to oncogene-induced senescence. Oncogene 27: 2801–2809 18193093 10.1038/sj.onc.1210950

[bib9] Franken NAP, Rodermond HM, Stap J, Haveman J, van Bree C (2006) Clonogenic assay of cells *in vitro*. Nat Protoc 1: 2315–2319 17406473 10.1038/nprot.2006.339

[bib10] Fung KL, Cheung HW, Wong HL, Yuen HF, Ling MT, Chan KW, Wong YC, Cheung AL, Wang X (2007) MAD2 expression and its significance in mitotic checkpoint control in testicular germ cell tumour. Biochem Biophys Acta 1773: 821–832 17467818 10.1016/j.bbamcr.2007.03.014

[bib11] Hampel B, Malisan F, Niederegger H, Testi R, Jansen-Durr P (2004) Differential regulation of apoptotic cell death in senescent human cells. Exp Gerontol 39: 1713–1721 15582287 10.1016/j.exger.2004.05.010

[bib12] Harley CB, Futcher AB, Greider CW (1990) Telomeres shorten during aging of human fibroblast. Nature 345: 458–460 2342578 10.1038/345458a0

[bib13] Hayflick L (1965) The limited *in vitro* life time of human diploid cell strains. Exp Cell Res 37: 614–636 14315085 10.1016/0014-4827(65)90211-9

[bib14] Kuilman T, Peeper DS (2009) Senescence-messaging secretome; SMS-ing cellular stress. Nat Rev Cancer 9(2): 81–94 19132009 10.1038/nrc2560

[bib15] Lengauer C, Kinzler K W, Vogelstein B (1997) Genetic instability in colorectal cancer. Nature 386: 623–627 9121588 10.1038/386623a0

[bib16] Lengauer C, Kinzler K W, Vogelstein B (1998) Genetic instability in human cancer. Nature 396: 643–649 9872311 10.1038/25292

[bib17] Li Y, Benezra R (1996) Identification of a human mitotic checkpoint gene: hsMAD2. Science 274: 246–248 8824189 10.1126/science.274.5285.246

[bib18] Ma W, Zhang D, Hou Y, Li YH, Sun QY, Sun XF, Wang WH (2005) Reduced expression of MAD2 BCL2 MAP kinase activity in pig oocytes after *in vitro* aging are associated with defects in sister chromatid segregation during meiosis II embryo fragmentation after activation. Biol Reprod 72: 373–383 15469999 10.1095/biolreprod.104.030999

[bib19] McGrogan BT, Gilmartin B, Carney DN, McCann A (2008) Taxanes, microtubules and the chemoresistant breast cancer. Biochim Biophys Acta 1785: 96–132 18068131 10.1016/j.bbcan.2007.10.004

[bib20] Percy MJ, Myrie KA, Neeley CK, Azim JN, Ethier SP, Petty EM (2000) Expression and mutational analysis of the human MAD2L1 gene in breast cancer cells. Genes Chromosomes Cancer 29: 356–362 11066082 10.1002/1098-2264(2000)9999:9999<::aid-gcc1044>3.0.co;2-n

[bib21] Rajaraman R, Guernsey DL, Rajaraman MM, Rajaraman SR (2006) Stem cells, senescence, neosis and self-renewal in cancer. Cancer Cell Int 8: 6–25 10.1186/1475-2867-6-25 PMC1664585 17092342

[bib22] Saunders W (2003) Bridging mitotic defects and clinical diagnoses. Cancer Biol Ther 2: 253–255 12878858 10.4161/cbt.2.3.379

[bib23] Shay JW, Roninson IB (2004) Hallmarks of senescence in carcinogenesis and cancer therapy. Oncogene 23: 2919–2933 15077154 10.1038/sj.onc.1207518

[bib24] Storchova Z, Pellman D (2004) From polyploidy to aneuploidy, genome instability and cancer. Nat Rev Mol Cell Biol 5: 45–54 14708009 10.1038/nrm1276

[bib25] Sudo T, Nitta M, Saya H, Ueno NT (2004) Dependence of Paclitaxel sensitivity on a functional spindle assembly checkpoint. Cancer Res 64: 2502–2508 15059905 10.1158/0008-5472.can-03-2013

[bib26] Swanton C, Tomlinson I, Downward J (2006) Chromosomal instability, colorectal cancer and taxane resistance. Cell Cycle 5(8): 818–823 16628000 10.4161/cc.5.8.2682

[bib27] Takahashi T, Haruki N, Nomoto S, Masuda A, Saji S, Osada H, Takahashi T (1999) Identification of frequent impairment of the mitotic checkpoint and molecular analysis of mitotic checkpoint genes, hsMAD2 and p55CDC, in human lung cancers. Oncogene 18: 4295–4300 10439037 10.1038/sj.onc.1202807

[bib28] To-Ho KW, Cheung HW, Ling MT, Wong YC, Wang X (2008) Oncogene 27: 347–357 17621272 10.1038/sj.onc.1210633

[bib29] Wang X, Jin DY, Ng RW, Feng H, Wong YC, Cheung AL, Tsao SW (2002) Significance of MAD2 expression to mitotic checkpoint control in ovarian cancer cells. Cancer Res 62: 1662–1668 11912137

[bib30] Wang X, Jin DY, Wong YC, Cheung AL, Chun AC, Lo AK, Liu Y, Tsao SW (2000) Correlation of defective mitotic checkpoint with aberrantly reduced expression of MAD2 protein in nasopharyngeal carcinoma cells. Carcinogenesis 21: 2293–2297 11133821 10.1093/carcin/21.12.2293

[bib31] Young AR, Narita M (2009) SASP reflects senescence. EMBO Rep 10(3): 228–230 19218920 10.1038/embor.2009.22 PMC2658552

[bib32] Yoon DS, Wersto RP, Zhou W, Chrest FJ, Garret ES, Kwon TK, Gabrielson E (2002) Variable levels of chromosomal instability and mitotic spindle checkpoint defects in breast cancer. Am J Pathol 161: 391–397 12163363 10.1016/S0002-9440(10)64194-6 PMC1850727

