# Cellular senescence induced by aberrant MAD2 levels impacts on paclitaxel responsiveness *in vitro*

**DOI:** 10.1038/sj.bjc.6605533

**Published:** 2010-01-19

**Authors:** M Prencipe, P Fitzpatrick, S Gorman, M Tosetto, R Klinger, F Furlong, M Harrison, D O'Connor, I B Roninson, J O'Sullivan, A McCann

**Correction to:** *British Journal of Cancer* (2009) **101,** 1900–1908; doi:10.1038/sj.bjc.6605419

Upon publication in Volume 101, Number 11, it was noted that the fourth author's name had been spelled incorrectly. We are now happy to correct this – the complete, correct author list is now shown above.

The authors would like to apologise for this mistake.

